# Prospective PED-study of intravitreal aflibercept for refractory vascularized pigment epithelium detachment due to age-related macular degeneration: morphologic characteristics of non-responders in optical coherence tomography

**DOI:** 10.1007/s00417-020-04675-y

**Published:** 2020-04-18

**Authors:** C. R. Clemens, F. Alten, J. Termühlen, N. Mihailovic, F. Rosenberger, P. Heiduschka, N. Eter

**Affiliations:** Department of Ophthalmology, University of Muenster Medical Center, Domagkstrasse 15, 48149 Muenster, Germany

**Keywords:** Age-related macular degeneration, Pigment epithelium detachment, Spectral-domain optical coherence tomography, Aflibercept, Anti-vascular endothelial growth factor

## Abstract

**Purpose:**

The aim of this study was to investigate the outcomes of a fixed intravitreal aflibercept regimen in patients with vascular pigment epithelium detachment (vPED) secondary to age-related macular degeneration with refractory subretinal fluid.

**Methods:**

A prospective, interventional case series involved 20 eyes of 20 patients with refractory subretinal fluid and vPED treated with at least three injections of intravitreal anti-VEGF prior to study inclusion. After study inclusion, patients were treated with three injections of intravitreal aflibercept 2 mg/0.05 mL monthly followed by injections every 8 weeks. Best-corrected visual acuity (BCVA) and spectral-domain optical coherence tomography (SD-OCT) were evaluated at all visits. Fluorescein angiography and indocyanine green angiography were performed at baseline and quarterly. Primary outcomes were effectivity of a fixed treatment as measured in change in BCVA, PED greatest linear diameter (GLD), and PED height from baseline to month 12. In an additional post hoc analysis, vPED patients were differentiated into two groups: (1) vPED lesions that showed persistence of subretinal fluid throughout 1 year of treatment and (2) vPED lesions that showed complete resolution of subretinal fluid at least at one of the monthly performed OCT volume scans. Reflectivity values were determined in the subretinal pigment epithelium (RPE) compartment in OCT scans at baseline, month 6 and 12.

**Results:**

A total of 18 patients completed the study protocol. The mean age was 74.8 ± 10.6 years, and six patients were female. The median BCVA of all patients was 72.0 ± 8.0 EDTRS letters at baseline and 72.5 ± 9.5 EDTRS letters at 12-month follow-up (*p* = 0.7420). The median PED height in all patients as measured in the OCT images significantly decreased from 372.0 ± 140.0 μm to 149.0 ± 142.0 μm after 12 months of treatment (*p* = 0.0020). Persistent subretinal fluid was present at every OCT control in six patients (group 1). Twelve patients showed resolution of subretinal fluid at least at one OCT control (group 2). Reflectivity values in the sub-RPE compartment in OCT scans were 41.48 ± 4.48 (group 1) and 42.62 ± 12.34 (group 2) at baseline (*p* = 0.854) and 65.88 ± 6.74 and 50.87 ± 14.11 at month 12 (*p* = 0.038).

**Conclusions:**

Intravitreal aflibercept in refractory vPED leads to a significant reduction in PED height and disease activity as well as preservation of BCVA over 1 year. Persistent subretinal fluid was present in PED lesions with high values of reflectivity under the RPE, suggesting both a diffusion barrier and an increasing fibrovascular maturization of the choroidal neovascularization.

**Trial registration:**

ClinicalTrials.gov Identifier: NCT03370380

## Introduction

Large prospective studies proved the efficacy of intravitreal anti-VEGF in the treatment of choroidal neovascularization (CNV) due to neovascular age-related macular degeneration (AMD) [1, 2]. However, with regard to define morphologic activity criteria in optical coherence tomography (OCT), a certain percentage of patients shows an inferior treatment response that has been subject of intense debate in the literature and is often referred to as “poor response,” “resistance,” or “tachyphylaxis” [3–5]. In this context, Guymer and colleagues recently discussed the origin of persistent subretinal fluid in OCT in exudative AMD patients. The authors suggest reasons for persistent subretinal fluid other than active exudation from a CNV membrane and question a zero-tolerance approach in certain AMD patients [6].

In the course of an anti-VEGF treatment, the morphologic OCT criterion of persistent subretinal fluid appears to be rather non-specific with regard to its exudative or non-exudative origin. Particularly in vascular pigment epithelium detachment (vPED) patients, treatment refractory subretinal fluid (SRF) is a common phenomenon [7]. Thus, a refinement of this morphologic activity criterion would greatly help to distinguish subretinal fluid of exudative origin that must be treated with anti-VEGF from subretinal fluid of non-exudative origin that should rather be observed.

In order to relieve the burden of a prolonged anti-VEGF therapy on patients’ quality of life and on health systems, clinical scientists are required to find ways to maximize the beneficial effects of anti-VEGF treatments and to minimize redundant injections. One way to achieve this goal is a continuous improvement of treatment regimen and a refinement of clinical treatment criteria.

The aim of this study was to investigate the functional and morphological outcomes of a fixed intravitreal aflibercept regimen in vPED patients with refractory subretinal fluid secondary to AMD. Additionally, this analysis focuses on morphologic differences between those vPED lesions that show resolution of subretinal fluid and those vPED lesions that show persistence of subretinal fluid throughout 1 year of treatment.

## Methods

This study was conducted as a prospective, single-arm, interventional, single-center study at the Department of Ophthalmology at the University of Muenster, Germany, between 2017 and 2018.

vPED lesions in 20 eyes of 20 patients were identified based on SD-OCT, fluorescein angiography (FA), and indocyanine green angiography (ICGA) as described elsewhere [8]. Inclusion criteria were a vPED lesion associated with CNV due to neovascular AMD and a best-corrected visual acuity (BCVA) of 24–73 letters according to the Early Treatment Diabetic Retinopathy Study (ETDRS) score. Patients had to be treated with at least three anti-VEGF injections prior to inclusion. Minimum vPED lesion height required for inclusion was ≥ 200 μm measured from Bruch’s membrane to the retinal pigment epithelium (RPE) reference in SD-OCT. Exclusion criteria included treatment-naïve lesions, subretinal hemorrhage, geographic atrophy, fibrovascular scar, pre-existing tear of the RPE, a discontinuous RPE layer in OCT, or highly reflective, laminar sub-RPE structures [9, 10].

At baseline, patients underwent clinical examination including BCVA using ETDRS charts, SD-OCT (OCT scan pattern, 61 B-scans, 30° × 25°; distance between B-scans, 119 μm), and FA and ICGA (30°×30°, 768 × 768 pixel) (Spectralis; Heidelberg Engineering, Germany). During follow-up, BCVA and SD-OCT (except the first and third visit) were evaluated at all visits. FA and ICGA were performed at baseline and quarterly. After study inclusion, patients were initially treated with three injections of intravitreal aflibercept (2 mg/0.05 mL) monthly followed by fixed injections every 8 weeks.

Primary outcome was the effectivity of a fixed treatment as measured in change in BCVA, PED greatest linear diameter (GLD), and PED height from baseline to month 12. PED height was measured as the vertical distance from Bruch membrane to the RPE border using the OCT scan revealing the most prominent lesion site. PED GLD was measured in the scan showing the maximum horizontal PED diameter. PED GLD was measured as the maximum distance from RPE elevation to RPE elevation.

**Fig. 1:**
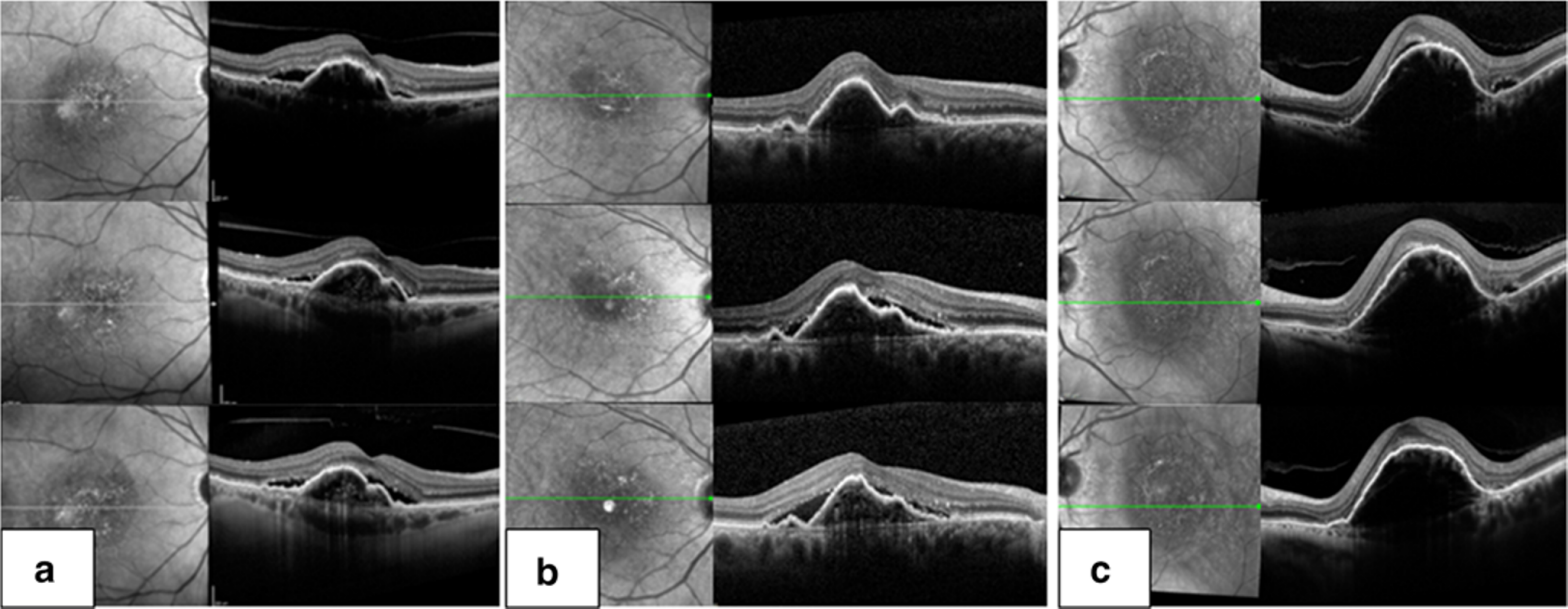
(**a** and **b**) Two exemplary patients of group 1 both with a vascular pigment epithelium detachment (vPED) lesion showing persistence of subretinal fluid throughout 1 year of treatment. Patient 1 (**a**) and patient 2 (**b**) first row baseline visit, second row 6-month visit, and third row 12-month visit baseline visit. Note the increase in hyperreflectivity in the sub-RPE space in the course of the treatment, while the subretinal fluid at the edge of the vPED lesion persists throughout the treatment. (**c**) Exemplary patient of group 2 with a vPED lesion showing complete resolution of subretinal fluid after the fourth injection. First row baseline visit, second row 3-month visit, and third row 4-month visit

In an additional post hoc analysis, vPED patients were differentiated into two groups: (1) vPED lesions that showed persistence of subretinal fluid at every monthly OCT volume scan throughout 1 year of treatment and (2) vPED lesions that showed complete resolution of subretinal fluid at least at one of the monthly performed OCT volume scans (Fig. 1).

**Fig. 2:**
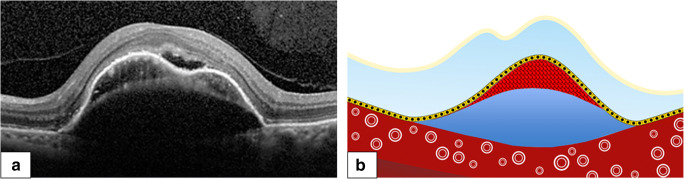
(**a**) Spectral-domain optical coherence tomography image of a vascular pigment epithelium detachment (vPED). Note the vascular (hyperreflective) component and the serous (hyporeflective) component in the subretinal pigment epithelium space. (**b**) Corresponding graphic to illustrate manual delineation of PED components: vascular component shown in red, and serous component shown in blue

Total reflectivity values and percentage of hyperreflective tissue in relation to serous fluid in the sub-RPE compartment in foveal OCT scans were determined at baseline, month 6 and 12. Image data of the foveal OCT scan were exported and further analyzed using Adobe Photoshop CS6 (Adobe Systems, Inc., CA, USA). The area of the sub-RPE space was delineated and converted into grayscale values attributing each pixel to a value that represents the signal strength. Within the area of the sub-RPE space, the vascular (hyperreflective) area and the serous (hyporeflective) area were separately delineated (Fig. 2). Two independent graders reviewed PED dimensions and manually delineated the area of the sub-RPE space, the vascular area, and the serous area.

### Statistical methods

Values are given as median ± median absolute deviation. *p* values were calculated within the groups (data paired) by Dunn’s multiple comparison test or the Wilcoxon test, and comparison between values of single time points in group 1 and group 2 was done by Mann-Whitney test (data not paired). The inter-grader agreements for PED dimensions were assessed using Bland-Altman statistics [11]. Statistical significance was set at *p* < 0.05.

## Results

A total of 18 patients completed the study protocol. The mean age was 74.8 ± 10.6 years, and 6 patients were female. The mean number of injections per patient was 7.4 during the study period. The mean number of injections per patient prior to study inclusion was 5.25 ± 0.49 (group 1, 4.9; group 2, 5.6). The median BCVA of all patients was 72.0 ± 8.0 EDTRS letters at baseline and 72.5 ± 9.5 EDTRS letters at 12-month follow-up. There was no change of BCVA in the total population (median change of BCVA in all patients after 12 months, + 1.5 ± 5.0 letters, *p* = 0.7420). The median PED height in all patients as measured in the OCT images significantly decreased from 372.0 ± 140.0 μm (group 1, 438.5 ± 156.0 μm; group 2, 316.0 ± 159.5 μm; *p* = 0.6454) at baseline to 195.8 ± 99.5 μm (group 1, 196.2 ± 106.0 μm; group 2, 168.5 ± 75.0 μm; *p* = 0.959) after 6 months and 149.0 ± 142.0 μm (group 1, 141.5 ± 141.5 μm; group 2, 178.5 ± 91.0 μm; *p* = 0.740) after 12 months of treatment. The median decrease in all patients after 12 months was 96.0 ± 122.5 μm (*p* = 0.002). The mean PED GLD in all patients decreased from 2091.5 ± 567.0 μm (group 1, 2328.0 ± 777.7 μm; group 2, 2091.5 ± 492.5 μm; *p* = 0.4418) at baseline to 1499.0 ± 487.0 μm (group 1, 1493.5 ± 535.0 μm; group 2, 1571.0 ± 432.5 μm; *p* = 0.7209) after 6 months and 1525.5 ± 542.5 μm (group 1, 1449.0 ± 1076.5 μm; group 2, 1568.5 ± 366.0 μm; *p* = 0.798) after 12 months of treatment. The median decrease in all patients after 12 months was 148.0 ± 634.5 μm (*p* = 0.0443).

Persistent subretinal fluid was present at every OCT control in six patients (group 1). Twelve patients showed resolution of subretinal fluid at least at one OCT control (group 2) (Fig. 1). Patients of group 2 showed complete resolution of subretinal fluid after 4.16 ± 1.9 injections. Eight out of 12 patients showed resolution after the first three injections. Ten patients remained without fluid during the rest of the follow-up period. In two patients, fluid reappeared after month 8, which was reabsorbed under further treatment.

If change of BCVA between baseline and at 12-months follow-up was compared within group 1 and group 2, no significant change was found either (*p* = 0.921 and *p* = 0.460, respectively). At both time points, BCVA was significantly better in group 2 (60.5 ± 14.5 in group 1 vs. 77.5 ± 3.5 in group 2, *p* = 0.025 at baseline, and 58.0 ± 12.5 in group 1 vs. 80.0 ± 3.0 in group 2, *p* = 0.014 at 12-month follow-up).

**Fig. 3:**
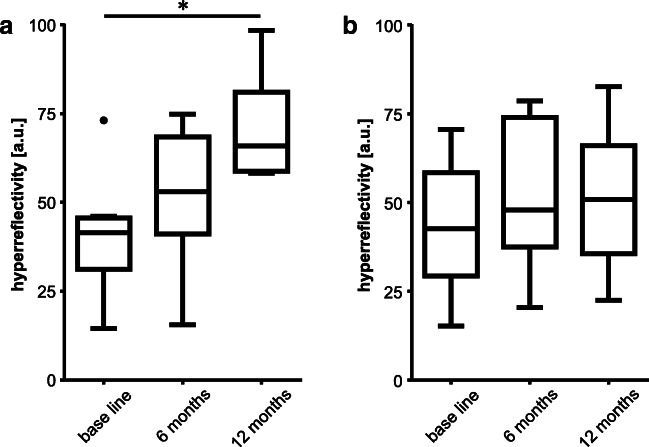
Box plot diagrams showing hyperreflectivity in the sub-RPE space (arbitrary units (au)) at baseline visit, 6 months and 12 months of (**a**) group 1 with those vPED lesions that show persistence of subretinal fluid throughout 1 year of treatment and (**b**) group 2 with those vPED lesions that show resolution of subretinal fluid within 1 year of treatment. *p < 0.05

Reflectivity values in the sub-RPE compartment in OCT scans were 41.48 ± 4.48 (group 1) and 42.62 ± 12.34 (group 2) at baseline (*p* = 0.854); 53.01 ± 11.74 and 47.94 ± 17.18 at month 6 (*p* = 0.777); and 65.88 ± 6.74 and 50.87 ± 14.11 at month 12 (*p* = 0.038). In group 1, reflectivity values increased significantly from baseline to month 12 (*p* = 0.037), whereas there was no significant change in group 2 over the period of observation (Fig. 3).

The percentages of hyperreflective tissue in relation to serous fluid in the sub-RPE compartment in foveal OCT scans were 40.24 ± 9.86% (group 1) and 41.35 ± 19.81% (group 2) at baseline (*p* = 0.7768); 48.02 ± 10.42% and 47.32 ± 16.79% at month 6 (*p* = 0.8541); and 60.97 ± 10.40% and 33.65 ± 7.79% at month 12 (*p* = 0.0148).

**Fig. 4:**
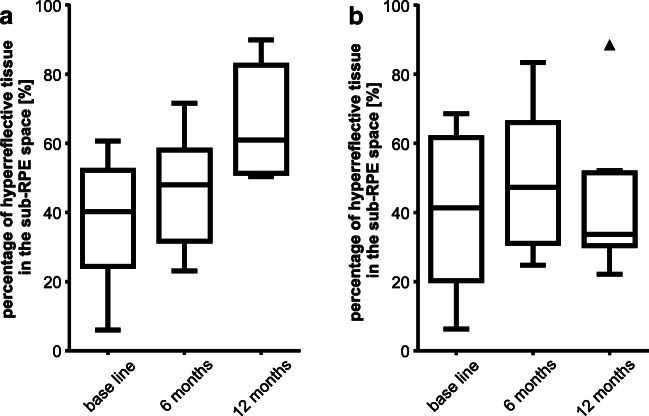
Box plot diagrams illustrating the percentage of hyperreflective tissue in the sub-RPE space (%) at baseline visit, 6 months and 12 months of (**a**) group 1 with those vPED lesions that show persistence of subretinal fluid throughout 1 year of treatment and (**b**) group 2 with those vPED lesions that show resolution of subretinal fluid within 1 year of treatment

In group 1, percentages of hyperreflective tissue in relation to serous fluid increased from baseline to month 12, though not significantly (*p* = 0.0733), whereas there was no apparent change in group 2 over the period of observation (Fig. 4). The two independent graders showed a good level of agreement in manually delineating PED dimensions. The mean difference and limits of agreement for percentages of hyperreflective tissue in relation to serous fluid in the sub-RPE compartment in foveal OCT scans at baseline, month 6, and month 12 were 0.12% (− 2.35 to 2.59%), − 0.28% (− 3.49 to 2.94%), and 0.01% (− 2.43 to 2.47%), respectively.

## Discussion

This study shows that intravitreal aflibercept in refractory vPED due to AMD leads to a significant reduction in vPED height as well as to preservation of BCVA over 1 year. Persistent subretinal fluid at the edge of the vPED lesion was present throughout the follow-up period in one third of patients, particularly in those showing high values of reflectivity in the vPED lesion’s sub-RPE space, suggesting both a diffusion barrier and an increasing fibrovascular maturization of the choroidal neovascularization.

Caution is warranted when interpreting sub-RPE reflectivity in OCT. For instance, increased hyperreflectivity of the underlying sub-RPE structures including PED content may occur in cases of beginning RPE atrophy. Therefore, a continuously intact RPE layer in OCT is required for analysis of sub-RPE reflectivity [9]. Secondly, highly reflective, multilayered, laminar sub-RPE structures have been described in OCT of vPED lesions that can typically be found in parallel alignment to Bruch’s membrane. The exhibited high reflectivity of this material is often as strong as the signal originating from the RPE and may also bias an analysis of sub-RPE reflectivity [10]. In our included patients, both morphologic criteria were not present. Thirdly, analyzed OCT scans were non-standardized regarding their reflectivity which is affected by multiple factors. Thus, OCT scan conversion into grayscales does not preclude any bias. Manual segmentation is always prone to errors. However, the comparison of graders shows a good agreement of the results in this study.

Previous studies on anti-VEGF in vPED patients analyzed the sub-RPE space in OCT scans. Punjabi et al. differentiated three types of vPED based on reflectivity of the material under the RPE on OCT: “hollow” with primarily hyporeflectivity under the RPE, “solid” with primarily hyperreflective signal under the RPE, and “mixed” with mixed reflectivity. In their retrospective study, hollow PEDs showed the best anatomical response, and solid lesions showed the poorest response [12]. Broadhead and co-workers qualitatively subclassified reflective properties according to the classification of Punjabi and similarly found that solid PEDs were less likely to experience reductions in PED height, width, and length under a 48-week aflibercept regimen [10].

In a study of intravitreal aflibercept in refractory vPED with or without subretinal fluid, Kim and colleagues used the same classification and additionally quantified the amount of subretinal fluid. They found that solid-type PEDs showed poorer improvements in BCVA, SRF volumes, and PED volumes compared with other types [13].

In our study, reflectivity of the sub-RPE space was assessed quantitatively. Notably, persistent subretinal fluid was present in vPED lesions with high values of reflectivity under the RPE suggesting both a diffusion barrier and an increasing fibrovascular maturization of the choroidal neovascularization. These quantitative findings are in line with qualitative imaging data of previous studies [10, 12, 13].

Undoubtedly, subretinal fluid in OCT associated with an active recent CNV due to neovascular AMD represents a clear activity sign and requires a relentless anti-VEGF treatment. Yet, in the course of the disease under continuative treatment, it becomes increasingly difficult to judge the CNV membrane’s exudative activity. Controversy exists regarding how to judge fluid-related OCT criteria in the further course of the disease [6]. Thus, additional biomarkers for actual exudative disease activity are required. With regard to vPED patients, persistent subretinal fluid in OCT despite regular anti-VEGF treatment over years is a well-known phenomenon [7]. Face to strict morphologic retreatment criteria, such OCT findings require further anti-VEGF injections. However, subretinal fluid in such patients may possibly result from a non-exudative process. Alternative explanations for persistent subretinal fluid and consequently alternative treatment options must be discussed.

Inactivity of occult CNV membranes, as seen on structural OCT scans, is usually not accompanied by a full regression of former active CNV. In the course of a continuative anti-VEGF therapy, fluid in the sub-RPE space resorbs, and the CNV remodels and eventually reaches a higher level of vascular maturity. Such inactive mature vessels are non-leaking, un-fenestrated, and covered by pericytes and may act as a diffusion barrier counteracting the resorption of subretinal fluid [14, 15]. At this stage, intravitreal anti-VEGF can hardly induce resorption of subretinal fluid as these vessels are only poorly accessible to antibodies. Similar findings were reported for pathologic neovascularization in malignant diseases [16, 17].

The often exactly identical quantity of subneurosensory fluid in monthly OCT scans is difficult to reconcile with the concept of tachyphylaxis in the presence of an active exudative CNV. Tachyphylaxis, tolerance, and resistance are erroneously used synonymously in the ophthalmic literature [3, 4]. Nevertheless, if one of these three phenomena were present, one would rather expect an increase in subneurosensory fluid despite anti-VEGF therapy in all scenarios.

Previous studies on anti-VEGF therapy for CNV due to AMD revealed that some subretinal fluid may even be beneficial for RPE, photoreceptor viability, and visual outcome [18, 19]. In the case of subretinal fluid and uncertainty regarding the presence of an active exudative CNV, Lek and co-workers recommend a follow-up within 1 week after treatment to assess the drug’s maximum effect. According to the authors, these cases may be observed if no morphologic response is observed [20].

Transferred to the clinical routine, the authors propose the following procedure in the event of a vPED lesion with treatment refractory subretinal fluid: If subretinal fluid without foveal involvement at the margin of a vPED lesion remains stable under constant treatment over 6 months, anti-VEGF therapy should be paused under monthly OCT control as long as the amount of fluid remains stable. If subretinal fluid increases, further injections should be performed. A future prospective study must evaluate the functional and morphologic outcome of this therapeutic approach.

Persistent subretinal fluid in vPED in the presence of a presumably non-exudative CNV membrane should be considered in a more differentiated way, and persistent subretinal fluid should not result in physicians continuing to inject indefinitely because of the failure to resolve this subretinal fluid. OCT findings and strict activity OCT criteria alone should not dictate the decision to treat with anti-VEGF. In this regard, we may also have to acknowledge the diagnostic limits of structural OCT to the extent that in the presence of constant subretinal fluid at the edge of a vPED lesion, the question of disease activity cannot be answered with structural OCT alone.

The study is limited by several factors. First, the relatively small sample size and the follow-up interval of 12 months limit the data’s validity. Besides, included patients were quite heterogeneous with regard to the number of injections prior to study inclusion, anti-VEGF agent, and onset of disease. Furthermore, there was no control group. Determination of two groups of vPED patients and characterization of sub-RPE properties were done retrospectively. Differences in the prior treatment may cause an imbalance between the two groups. Yet, the total number of prior injections was not significantly different between the two groups. Lastly, characterization of sub-RPE reflectivity was performed in only that OCT scan that included the peak of the vPED lesion.

In conclusion, intravitreal aflibercept in refractory vPED due to AMD led to significant reduction in PED height as well as a preservation of BCVA over 1 year. Persistent subretinal fluid was present in vPED lesions with high values of reflectivity under the RPE suggesting both a diffusion barrier and a fibrovascular maturization of the choroidal neovascularization. In the future, a more differentiated consideration of the activity criterion of subretinal fluid in vPED due to AMD by means of enhanced imaging modalities seems reasonable, particularly with regard to an optimal anti-VEGF treatment strategy.

## References

[1] Heier JS, Brown DM, Chong V (2012). Intravitreal aflibercept (VEGF trap-eye) in wet age-related macular degeneration. Ophthalmology.

[2] Rofagha S, Bhisitkul RB, Boyer DS (2013). Seven-year outcomes in ranibizumab-treated patients in ANCHOR, MARINA, and HORIZON: a multicenter cohort study (SEVEN-UP). Ophthalmology.

[3] Binder S (2012). Loss of reactivity in intravitreal anti-VEGF therapy: tachyphylaxis or tolerance?. Br J Ophthalmol.

[4] Arjamaa O, Minn H (2012). Resistance, not tachyphylaxis or tolerance. Br J Ophthalmol.

[5] Hara C, Wakabayashi T, Fukushima Y, Sayanagi K, Kawasaki R, Sato S, Sakaguchi H, Nishida K (2019). Tachyphylaxis during treatment of exudative age-related macular degeneration with aflibercept. Graefes Arch Clin Exp Ophthalmol.

[6] Guymer RH, Markey CM, McAllister IL, Gillies MC, Hunyor AP, Arnold JJ, FLUID Investigators (2019). Tolerating subretinal fluid in Neovascular age-related macular degeneration treated with ranibizumab using a treat-and-extend regimen: FLUID study 24-month results. Ophthalmology.

[7] Gianniou C, Dirani A, Jang L, Mantel I (2015). Refractory Intraretinal or subretinal fluid in neovascular age-related macular degeneration treated with intravitreal ranizubimab: functional and structural outcome. Retina.

[8] Clemens CR, Bastian N, Alten F, Milojcic C, Heiduschka P, Eter N (2014). Prediction of retinal pigment epithelial tear in serous vascularized pigment epithelium detachment. Acta Ophthalmol.

[9] Clemens CR, Krohne TU, Charbel Issa P (2012). High-resolution optical coherence tomography of subpigment epithelial structures in patients with pigment epithelium detachment secondary to age-related macular degeneration. Br J Ophthalmol.

[10] Broadhead GK, Hong T, Zhu M (2015). Response of pigment epithelial detachments to intravitreal aflibercept among patients with treatment-resistant neovascular age-related macular degeneration. Retina.

[11] Bland JM, Altman DG (1986). Statistical methods for assessing agreement between two methods of clinical measurement. Lancet.

[12] Punjabi OS, Huang J, Rodriguez L (2013). Imaging characteristics of neovascular pigment epithelial detachments and their response to anti-vascular endothelial growth factor therapy. Br J Ophthalmol.

[13] Kim K, Es K, Kim Y, Yang J, Yu S, Hw K (2019). Outcome of intravitreal aflibercept for refractory pigment epithelial detachment with or without subretinal fluid and secondary to age-related macular degeneration. Retina.

[14] Helfrich I, Scheffrahn I, Bartling S (2010). Resistance to antiangiogenic therapy is directed by vascular phenotype, vessel stabilization, and maturation in malignant melanoma. J Exp Med.

[15] Pachydaki SI, Jakobiec FA, Bhat P (2012). Surgical management and ultrastructural study of choroidal neovascularization in punctate inner choroidopathy after bevacizumab. J Ophthalmic Inflamm Infect.

[16] Sarks JP, Sarks SH, Killingsworth MC (1997). Morphology of early choroidal neovascularisation in age-related macular degeneration: correlation with activity. Eye.

[17] Lu C, Thaker PH, Lin YG (2008). Impact of vessel maturation on antiangiogenic therapy in ovarian cancer. Am J Obstet Gynecol.

[18] Waldstein SM, Wright J, Warburton J, Margaron P, Simader C, Schmidt-Erfurth U (2016). Predictive value of retinal morphology for visual acuity outcomes of different ranibizumab treatment regimens for neovascular AMD. Ophthalmology.

[19] Sharma S, Toth CA, Daniel E (2016). Macular morphology and visual acuity in the second year of the comparison of age- related macular degeneration treatments trials. Ophthalmology..

[20] Lek JJ, Caruso E, Baglin EK (2018). Interpretation of subretinal fluid using OCT in intermediate age-related macular degeneration. Ophthalmol Retina.

